# American Medical Association Section on Oral and Dental Surgery

**Published:** 1882-08

**Authors:** 


### ARTICLE III.

### *American Medical Association.*

#### SECTION ON ORAL AND DENTAL SURGERY.

Dr. D. H. Goodwillie, of Mew York, Chairman.

Dr. Truman G. Brophy, of Chicago, Secretary.

TUESDAY, JUNE 6TH—FIRST DAY.

The Chair appointed Drs. Allport, Brophy, and Williams, sub-committee, to which all papers read before the section should be referred.

Dr. William D. Kempton, of Cincinnatti, presented a paper on "Oral Hygiene," of which the following is a synopsis:

From the beginning the sole aim of practitioners was to discover a cure for disease and, although sometimes harmful, the medication received the credit. Now, one of the main objects of practitioners is to prevent disease. The profession is indebted to specialists for many, if not most, of these discoveries, as they are more apt to arrive at definite results than those whose attention is occupied with the whole field of medicine.

Oral hygiene was very important, and the evils arising from the neglect of it were far reaching. Good, healthy, even strong teeth were beautiful as well as serviceable, and indicated care. But when we find one whose teeth resemble the charred trunks of stately trees after the fiery scourge has visited the forest, and whose breath is suggestive of a cess-pool, we ask, whence comes this sad havoc?

Dr. Kempton then entered into a careful analysis of the teeth giving their elements and functions, and the acid theory of decay by Dr. George Watt, with the factor that modifies the action of acids, viz., vitality. Then followed a list of the evils resulting from diseased teeth, showing that the whole system was more or less affected and deranged; many of the cases of headache, earache, affections of the eye, and stomach, being traceable oftentimes to badly decayed teeth.

The doctor closed his paper with directions for preventing decay in teeth, prefacing with the remark that those teeth in which decay had already set in should be either extracted or filled. Physicians should feel it their duty to point out to their patients the results of neglect of the teeth, and no medical school should consider its corriculum complete unless some attention is paid to the teeth.

### PHOSPHATES IN FOOD.

The paper was discussed at length by Drs. Williams, Talbot, Allport, Lawrence, Marshall, Goodwillie, Ellmer, Reed and, others, during which the subject of Phosphates in Food was introduced, The enamel of the teeth is composed mainly of phosphate of lime, and Dr. Allport was very earnest when he made the remark that our food should be taken as nearly as possible in the condition in which God prepared it. He referred especially to wheat, asserting that the so-called patent process of making flour eradicated much of the phosphate in the wheat, the result being that not enough of this was left to keep the teeth strong and healthy. He did not advocate the use of phosphates alone, but he did protest against food which had not enough of them to keep the system and teeth in perfect order.

Dr. Lawrence antagonized the stress placed upon this subject, alleging that other elements were as necessary as phosphates.

The Section then adjourned to meet at 3 p. m. Wednesday.

### Wednesday, June 7th—Second Day.

### HEREDITY IN DENTAL DEVELOPMENT.

Dr. W. C. Barrett, of Buffalo, briefly sketched a case which had come under his observation, and which he illustrated by plaster casts, showing the persistence of heredity in dental development.

Dr. J. S. Marshall, of Syracuse, N. Y., then read a paper on "The Need of Dental and Oral Surgeons in the Army and Navy." The following is a synopsis:

This subject has often been brought to the attention of the profession, but never formally till August, 1861, at the American Dental Convention in New Haven, where it was referred to a committee of five, who, after consultation with Surgeon-General Hammond, reported favorably to the appointment of dental and oral surgeons in the army and navy. In 1868 Senator Hamlin introduced a bill before Congress providing for the appointment of such surgeons in the army and navy, but this measure died before it was born. The second attempt was made during the Forty-second Congress by Representative Townsend, who merely advocated the appointment of such surgeons to the military and naval academies, but nothing came of this but the appointment of a dental surgeon at the naval academy at Annapolis with the rank of assistant surgeon. Dr. Marshall then stated the necessity existing for such appointments.

The government provides for the care of a soldier in all cases, except where his teeth are concerned. Soldiers on the frontier and sailors on a long cruse, have no opportunity of receiving dental services, no matter how much they may need such attention, and the disease must run its course, being turned over to the bungling of the hospital steward or some less competent person. The treatment of fractures or gunshot wounds of the lower jaw, is the same as twenty-five or thirty years ago, being much behind the times. The interdental splint, invented by Dr. J. B. Bean, of Georgia, during the civil war, and improved by Dr. Norman W. Kingsley, of New York, is a very great improvement in the treatment of these cases, and is endorsed by the best surgeons the world over. The success with this splint has been remarkable.

The objection to the appointment of dental and oral surgeons in the army and navy, is that the amount of oral diseases is so small as not to require specially educated surgeons to treat them. Dr. Marshall then gave some statistics showing the relative number of men in the army and navy who, in the years 1878 and 1879, were reported as having needed dental services, also the opinions of the surgeons general of the army and navy, General Hancock and Admiral Porter, relative to the appointment of such surgeons. To petition Congress for action is useless, without the heads of medical departments see the need and make the recommendation. The paper closed with a recommendation that a committee be appointed by this Section to arrange a blank statistical report covering all the dental and oral diseases, and request the surgeons general of the army and navy to incorporate them in the regular medical and surgical reports.

A lively discussion followed on the subject of the paper, which resulted in the offering of a resolution by Dr. Allport that a committee of three be appointed by the Section, who, in connection with Dr. Maynard, of Washington City, and the surgeons general of the army and navy, should make what efforts they deemed advisable regarding the appointment of dental and oral surgeons in the army and navy, and to report to the association next year. This motion was adopted, and Drs. Allport, Marshall, and Williams were appointed such committee.

### FOOD IN ITS RELATION TO THE DEVELOPMENT OF TISSUES.

Dr. Lawrence, of New York city, presented a resolution calling for the appointment of a committee to consider the subject of food, including mastication, insalivation, digestion, and assimilation, in its relation to the development of the different tissues and organs of the body.

Drs. Lawrence, Talbot, and Kempton were appointed as the committee.

The Section then adjourned to meet Thursday at 3 p. m.

THUESDAY, JUNE 8TH—THIRD DAY.

Dr. Goodwillie called attention of the Section to cases of Necrosis from Arsenic, and illustrated them with wax models.

Case I. Showed by two models, necrosis of lower jaw from each ramos forward. The case, before and after, with a new deposit of bone without any deformity. Photograph of the patient also shown.

Case II.—Two models showing a case of poison by arsenic and necrosis of right superior maxillary.

*a.* Showing case one week after removal of necrosed bone; without, in the least, disturbing the soft tissue; also showing the formation of new bone.

*b.* The new bone complete and the mouth perfect; and no external deformity.

Case III.—Upper maxillary showing abscesses formed at nearly all the teeth, the result of applying arsenic to destroy sensibility of the dentine before filling the teeth.

The above served to show the sad results of the improper use of this powerful agent in devitalizing dental pulps.

Dr. Eugene S. Talbot, of Chicago, then read a paper on "The Injurious Effects of Mercury as Used in Dentistry." The paper was confined to the use of amalgam fillings in natural teeth.

"There can no longer be any doubt that amalgam fillings in teeth will sooner or later produce mercurial poisoning. The dire effects of this metal are not always seen immediately after the fillings are inserted, years sometimes elapsing before the injurious effects are felt and noticed."

The history of two well-marked cases were given by Dr. Talbot, the persons affected having called upon him for treatment. The amalgam fillings were removed, and gutta-percha temporarily substituted, these in turn being replaced with gold, after which all symptoms of mercurial poisoning disappeared. A detailed account of a series of experiments made by him were then presented, the conclusions and results being as follows:

*First.* Mercurial vapor is given off from amalgam fillings at all ages and from all varieties, even from fillings sixteen years old, the vaporization being sufficient in quantity to respond to chemical tests.

*Second.* Minute doses of mercury, if taken internally three times a day, are capable of producing decided effects.

*Third.* Mercury when inhaled into the lungs is far more active than when taken into the stomach.

*Fourth.* If small doses taken into the stomach occasionally are capable of producing marked effects, and the vapor is much more active than the solid preparations of the metal, is it not a necessary consequence that amalgam fillings which are constantly giving off mercurial fumes to be inhaled into the lungs, not a few times daily, but always, without cessation, day or night, do, in many sensitive persons, produce deleterious effects?

*Fifth.* When tons of this material are consumed annually, is it not credible that many constitutions are affected?

*Sixth.* Physicians in treating dyspeptics, anæmics, and persons suffering from nervous debility, would do well to examine the mouths of patients and know if artificial teeth on red rubber or fillings of natural teeth have in their composition mercury or any of its compounds.

The subject, "How Dentists should be Educated," was presented by Dr. W. W. Allport, of Chicago in a paper of considerable length. The introduction was a review of dental surgery—it, with all other branches of medicine, having emanated from a common center, and the possibility was that in the future these diverse systems would more and more tend to consolidate.

Dr. Allport then said that the dental and oral surgeon must be educated both in mechanical dentistry and oral surgery, for no disease can be intelligently treated without a knowledge of the histology, anatomy, and the physiology of the organ or organs diseased, as well as the pathology prognosis and rationale of the treatment employed to restore the parts to a healthy condition; and this is medical science. The successful oral surgeon must have a thorough medical education in all its branches, supplemented by special instruction in dental surgery. Over forty years ago Drs. Harris, Hayden, and others, sought to establish a department for teaching dental and oral surgery in the medical department in the University of Maryland; but their application was refused. Dr. Harris, however, succeeded in organizing what was known as the Baltimore College of Dental Surgery, and the graduates of this institution were given the degree of D. D. S.—doctor of dental surgery. In addition to this, several medical colleges have been induced to establish dental schools in connection with their medical departments, but as yet none of these institutions have required a full medical education of their dental graduates.

All dental and oral surgeons should receive a medical education and become legitimate specialists in its practice, and all medical graduates should be as fully educated in diseases of the teeth and the science of their treatment as they are in other diseases.

Dr. J. B. Lawrence, of New York, then read a paper on "Medico-Dental Science," after which the Section adjourned.—*Medical Record.*

